# Innovative BPPO Anion Exchange Membranes Formulation Using Diffusion Dialysis-Enhanced Acid Regeneration System

**DOI:** 10.3390/membranes11050311

**Published:** 2021-04-23

**Authors:** Muhammad Imran Khan, Majeda Khraisheh, Fares AlMomani

**Affiliations:** 1 Research Institute of Sciences and Engineering, University of Sharjah, Sharjah 27272, United Arab Emirates; emran@mail.ustc.edu.cn or; 2 Department of Chemical Engineering, College of Engineering, Qatar University, Doha 2713, Qatar; falmomani@qu.edu.qa

**Keywords:** diffusion dialysis, triphenylphosphine, BPPO, anion exchange membrane, ion exchange capacity, water uptake

## Abstract

Recycling of acid from aqueous waste streams is crucial not only from the environmental point of view but also for maturing the feasible method (diffusion dialysis). Anion exchange membrane (AEM)–based diffusion dialysis process is one of the beneficial ways to recover acid from aqueous waste streams. In this article, the synthesis of a series of brominated poly (2, 6–dimethyl-1, 4–phenylene oxide) (BPPO)-based anion exchange membranes (AEMs) through quaternization with triphenylphosphine (TPP) were reported for acid recovery via diffusion dialysis process. The successful synthesis of the prepared membranes was confirmed by Fourier transform infrared (FTIR) spectroscopy. The as-synthesized anion exchange membranes represented water uptake (W_R_) of 44 to 66%, ion exchange capacity of (IEC) of 1.22 to 1.86 mmol/g, and linear swelling ratio (LSR) of 8 to 20%. They exhibited excellent thermal, mechanical, and acid stability. They showed homogeneous morphology. The acid recovery performance of the synthesized AEMs was investigated in a two compartment stack using simulated mixture of HCl and FeCl_2_ as feed solution at room temperature. For the synthesized anion exchange membranes TPP–43 to TPP–100, the diffusion dialysis coefficient of acid (U_H_^+^) was in the range of 6.7 to 26.3 (10^−3^ m/h) whereas separation factor (S) was in the range of 27 to 49 at 25 °C. Obtained results revealed that diffusion dialysis performance of the synthesized AEMs was higher than the commercial membrane DF–120B (U_H_^+^ = 0.004 m/h, S = 24.3) at room temperature. It showed that the prepared AEMs here could be excellent candidates for the diffusion dialysis process.

## 1. Introduction

Nowadays, a large quantity of acid waste has been generated by many industries [1,2]. These acids are a byproduct of various processes such as leaching, fine-tuning of metals, and mining activities [3]. This waste has become a crucial candidate for generating water pollution, thus resulting in huge economical losses [4]. Therefore, several works were carried out to discover such a type of technology that can remove these acids and wastes from wastewater such as neutralization, fluidized beds, and precipitation [3,5]. Several techniques in the field of membrane technologies were utilized for acidic and alkaline recovery, including several individual processes such as electrodialysis, nanofiltration, or the most preferred process called diffusion dialysis [6,7,8,9,10].

**Figure 1:**
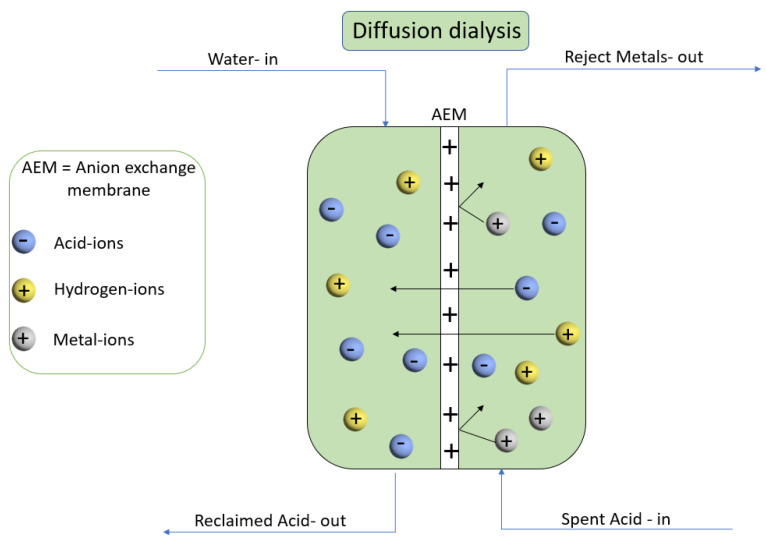
Schematic diagram for the procedure of diffusion dialysis.

The diffusion dialysis (DD) system due to its superiority compared to other methods is installed for acid extraction since it is a green process that requires no power dissipation and has simple progression and equipment [5]. Initiated by Graham (1861), the notion of dialysis demonstrates a concentration driven spontaneous process using a semi-permeable membrane to split large and small molecules from each other [11]. However, this method has some limitations such as it needs a crucial fabrication of such an ion exchange membrane that can permit the ions to permeate through it as well as some equilibrium restraints to the concentration of the solution that is regenerated and stimulated [12,13]. Generally, diffusion dialysis membranes are divided into two categories, they either fall into cation exchange membranes (CEM) or AEM. The AEMs stand as an essentially used method for regeneration and separation of the acids whilst CEMs are for the separation of bases. AEMs are subjected to more consideration since recovery of acid has more demand than bases, so countless AEMs are in the development and manufacturing phases. The consequences and outcomes for the physical and chemical properties of membranes on the diffusion dialysis process are also a hot topic for researchers these days and excellent outcomes have been reported as per studies [14,15,16,17,18]. Several pieces of research demonstrate and indicate that the anion exchange membrane is the key component and efficient process for acid recovery. It is associated with its high ability to separate and regenerate the acid from wastewater. Poly(DMAEM–co–γ–MPS) gave a diffusion dialysis coefficient U_H_^+^ of 0.016–0.029 m/h and a separation factor S of 23.3–87.7 [19]. Quaternized poly(2,6–dimethyl-1,4–phenylene oxide) membranes displayed an ion exchange capacity value of 2.07 mmol/g, diffusion coefficient U_H_^+^ of 0.020 m/h, and a separation factor S of 73 at room temperature [20]. Highly charged hierarchical porous membranes increased the performance of diffusion dialysis with higher diffusion dialysis coefficient U_H_^+^ of 0.0273 m/h and a separation factor S of 86.5 at 25 °C [21]. A diffusion dialysis coefficient U_H_^+^ of 0.019 m/h and a separation factor S of 127 was reported for series-connected hexacations cross-linked membranes [22]. Diffusion dialysis as shown in (Figure 1) has prominence in industries that are fundamentally established on metal processing because their acid can be recovered and regenerated concurrently with endorsing the recycling of the metal ions from waste streams [13].

The regeneration competence for acid in the diffusion dialysis process entails comparatively superior performance for anion exchange membranes. Chiefly, the movement for anions is through AEM, because of the concentration difference that is the main driving force during the diffusion dialysis process. This is because of the electrostatic association of cationic units in the polymer frameworks and architecture. Consecutively, through the anion exchange membrane, the metallic and H^+^ ions are supplemented due to the electroneutrality [14]. Thus, the source of the challenge is the division between H^+^ and metal ions. Particularly, in comparison to H+, the metal ions traditionally exhibit greater hydrated ionic radii and supplementary charges. Due to this, the main conditions to be explored for evolving AEMs with enhanced efficiency for diffusion dialysis are the size prohibition effect and electrostatic repulsion.

**Figure 2:**
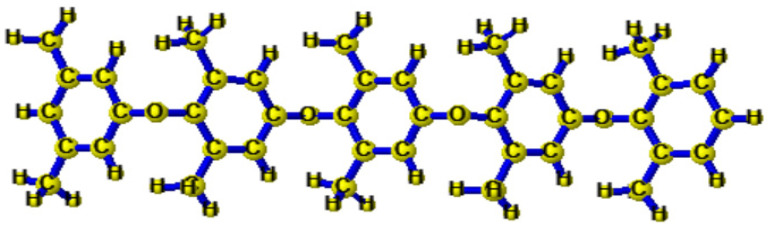
Three-dimensional structure of poly (2,6–dimethylene–1,6–phenylene oxide) (PPO).

**Figure 3:**
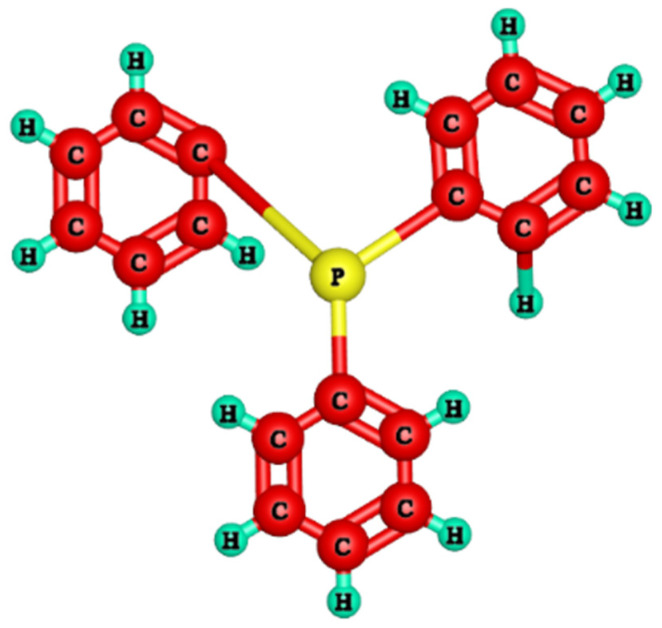
Three-dimensional structure of triphenylphosphine (TPP).

Until now, multiple methods have been developed to produce anion exchange membranes that accommodate the diffusion dialysis performance specifications, including modifying the polymer architectures and altering the cationic head groups. The polymer structures of the anion exchange membrane are essential for their characteristics. The anion exchange membranes should be water-insoluble and mechanically significant to achieve optimum stability and selectivity. Previously, numerous polymer architectures including poly (arylene ether ketone) [23,24], poly(arylene ether sulfone) [25,26,27], poly(fluorenyl ether ketone sulfone) [28], poly(vinyl alcohol) [29], poly(2,6–dimethyl–1,4–phenylene oxide) [30,31], and poly(ether–imide) [32] were established for the manufacture of AEMs (anion exchange membranes). Besides, to replicate well-connected ion-conducting pathways, the efficiency and capacity of ion exchange membranes should be high, leading to a higher value of diffusion dialysis coefficient for anion exchange membranes [33]. Poly (2,6–dimethylene–1,6–phenylene oxide) (PPO, Figure 2)) was fundamentally utilized due to its superior mechanical properties and membrane developing success amongst reported polymer architectures. The use of BPPO for anion exchange membrane formation restricted any use of chloromethyl ether (CME), which is a hazardous and restricted material [34]. It is indeed easy to modify its hydrophobic surface to one that is hydrophilic. Thus, in the presented work, BPPO has been used as a polymer architecture for these purposes. Contrarily, the cationic head groups attached to the anion exchange membrane polymer architecture that investigated anion permeability also have been revealed. For the anion exchange membranes, a good cationic head group should possess benefits such as high absorbency of anion, superficial procedure for synthesis, and last but not the least, low costs for the preparatory materials. In the studies performed until now, several AEMs owning different cationic head groups have been fabricated for the diffusion dialysis process [20,35,36,37,38,39,40]. Nevertheless, the diffusion dialysis process for these anion exchange membranes is not adequate. Hence, this study will fabricate anion exchange membranes for the diffusion dialysis process by utilizing a new functional group called triphenylphosphine (Figure 3) (TPP). Triphenylphosphine encompasses a lower acidity value (pKa = 7.64), resulting in building the anions such that they have much easier disassociation with respect to the functional groups, resulting in higher penetrability of anions [36]. Furthermore, it is the first attempt to utilize triphenylphosphine as a cation head group for the formulation of anion exchange membranes for diffusion dialysis applications, applying the best knowledge and techniques possible.

In this paper, the formulation of a sequence of homogeneous anion exchange membranes based on BPPO with varying physico–chemical characteristics has been revealed by altering the amount of triphenylphosphine (shown in Figure 3) through the use of the solution casting method into the polymer matrix. This formulation of anion exchange membranes has been validated by FTIR spectroscopy. A detailed investigation was carried out on the ion exchange power, water absorption, linear swelling ratio, morphology, chemical, mechanical, and thermal stability of the produced anion exchange membranes. In particular, through diffusion dialysis, the acid recovery reliability of synthesized anion exchange membranes was exposed and contrasted with the commercial membrane DF-120B at 25 °C.

## 2. Experimental

### 2.1. Materials 

Sigma-Aldrich chemicals (St. Louis, MO, USA) supplied poly (2,6–dimethyl–1,4–phenylene oxide) (PPO). Other materials that were used included 2,2–Azo–bis–isobutyro nitrile (AIBN), triphenylphosphine (TPP), chlorobenzene, ethanol, chloroform, N-methyl–2–pyrrolidone (NMP), sodium chloride (NaCl), N–Bromo–succinimide (NBS), silver nitrate (AgNO_3_), potassium chromate (K_2_CrO_4_), sodium sulfate (Na_2_SO_4_), hydrochloric acid (HCl), ferrous chloride (FeCl_2_.4H_2_O), and methyl orange (MO) that were received from Sinopharm Chemical reagent Co. Ltd. (Shanghai, China) and were utilized as attained. Moreover, deionized water was utilized throughout this research work. 

### 2.2. Procedure for Synthesis of BPPO

According to the previously reported and testified method, this study describes the successful synthesis of BPPO [41]. First, 6 g of PPO (50 mmol) was dissolved initially into chlorobenzene (50 mL) in a round bottom flask possessing a magnetic stirrer and refluxed condenser. The solution was stimulated steadily in order to reduce the variance in internal concentrations with the movement of components through the membrane [42]. To the above-stirred solution of PPO, NBS (4.45 g, 25 mmol) and AIBN (0.25 g, 1.5 mmol) were added. The attained reaction mixture was heated at 135 °C for 3 h. The reaction mixture was poured into an excess of ethanol to precipitate the product after cooling at room temperature. The attained solid was filtered and washed with ethanol, and the residue subsequently re-dissolved into chloroform (60 mL) and precipitated into ethanol solution. The attained solid was collected as a light-yellow powder and dried under vacuum for 48 h at 40 °C to get BPPO with a bromination ratio of 75% (DB = 0.75).

### 2.3. Procedure for Synthesis of Anion Exchange Membranes 

**Figure 4:**
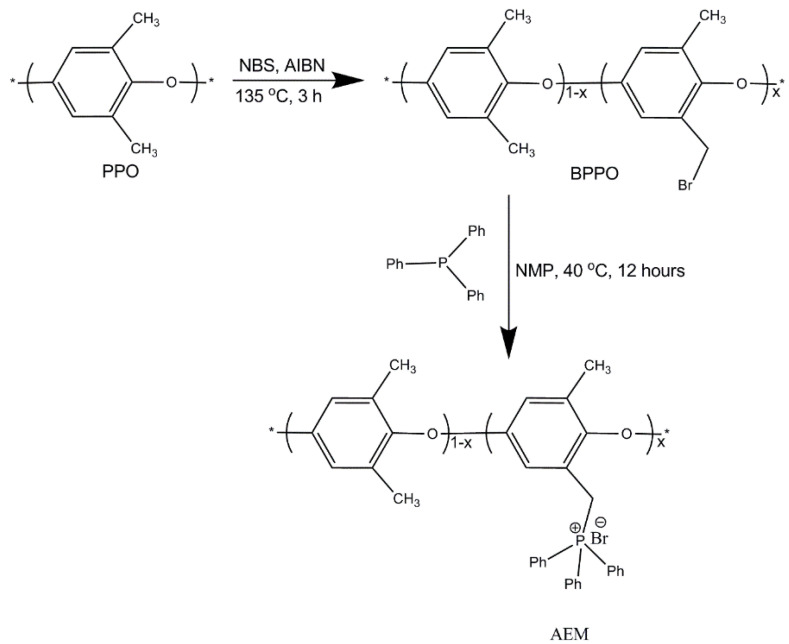
The synthesis of BPPO-based AEMs.

In this work, the solution casting method was utilized for the synthesis of BPPO-based anion exchange membranes as reported in our previous research [35,43,44,45,46,47]. At first, 7% (wt %) solution was formed by dissolving 0.7 g (2.6 mmol) of BPPO into N-methyl-2-pyrrolidone (NMP) solvent. Anion exchange membranes with different physico–chemical properties were obtained by adding 0.30, 0.50, and 0.70 g (1.10, 1.90, and 2.60 mmol) of TPP into the casing solution. To accelerate the reaction, the reaction mixture was stirred at 40 °C for 12 h. Then, the solution was casted onto a glass plate at 60 °C for 24 h. Dried anion exchange membranes were peeled off from glass plates and washed with deionized water. The prepared anion exchange membranes were denoted as TPP–43, TPP–71, TPP–100, where 41, 71, and 100 refer to the percentage of TPP to BPPO. Figure 4 represents the chemical structure of the synthesized AEMs.

### 2.4. Characterizations

#### 2.4.1. H NMR and FTIR Analysis

The 1H NMR (DMX 300 NMR spectrometer operating at 300 MHZ) was utilized to confirm the successful formulation of BPPO. The successful synthesis of the AEMs was proved by attenuated total reflectance (ATR) with FTIR spectrometer (Vector 22, Bruker, Lakeland, FL, USA) in the range of 4000–400 cm^−1^. 

#### 2.4.2. Ion Exchange Capacity, Water Uptake, and Linear Swelling Ratio

The classical Mohr’s method was utilized to determine the ion exchange capacity of the synthesized anion exchange membranes as reported in our previous work [35,43,44]. For the conversion of bromide form to chloride, the membrane samples were immersed into 1.0 (M) NaCl solution for 2 days. To eliminate excessive NaCl, the membrane samples were washed with distilled water. Then, the membranes samples were immersed in 0.5 (M) Na_2_SO_4_ solutions for 2 days. The quantity of Cl^–^ ions eliminated was measured by titration with 0.05 (M) AgNO_3_ utilizing K_2_CrO_4_ as an indicator. It was measured by the below relationship:

(1) $$\mathrm{IEC}=\frac{C_{\mathrm{AgNO}3}V_{\mathrm{AgNO}3}}{W_{\mathrm{Dry}}}$$

where *W*, *V*, and *C* depict the dry weight of the membrane sample, titer volume during titration, and the concentration of AgNO_3_ solution, respectively.

The dry and precisely weighed synthesized anion exchange membranes were soaked and saturated into the deionized water at room temperature. After removing surface water with a tissue paper, the wet weight of membranes was calculated. Water uptake (*W_R_*) was attained from the difference in mass before and after the drying of the membranes by the below equation [35,44]:

(2) $$W_{R}=\frac{W_{\mathrm{WET}}-W_{\mathrm{DRY}}}{W_{\mathrm{DRY}}}\times 100\%$$

where *W*_WET_ and *W*_DRY_ show, respectively, the wet and dry weights of the prepared anion exchange membranes.

The dried anion exchange membrane samples (3 × 1) cm^2^ were soaked in deionized water at room temperature. The wet length of membranes was measured after removing surface water with a tissue paper. The linear swelling ration was measured from the below formula [48]:

(3) $$\mathrm{LER}=\frac{L_{\mathrm{WET}}-L_{\mathrm{DRY}}}{L_{\mathrm{DRY}}}\times 100\%$$

where *L*_WET_ and *L*_DRY_ show wet and dry lengths of membrane samples, respectively.

#### 2.4.3. Thermal and Mechanical Stability 

Herein, a Shimadzu TGA–50H analyzer was utilized to reveal thermal stability of the synthesized AEMs with a heating rate of 10 °C/min under nitrogen flow within the temperature range 25 to 800 °C. Mechanical stability of the prepared AEMs was measured using Q800 dynamic mechanical analyzer (DMA, TA Instruments, Columbus, OH, USA) at a stretch rate of 0.5 N/min.

#### 2.4.4. Chemical Stability

For the synthesized anion exchange membranes, the chemical stability was investigated in terms of weight loss as a function of time by immersing them into HCl/FeCl_2_ solution at 60 °C. 

#### 2.4.5. Morphology

A field emission scanning electron microscope (FE-SEM, Sirion200, FEI Company Hillsboro, OR, USA) was utilized to investigate the structure of the prepared AEMs. 

#### 2.4.6. Diffusion Dialysis of HCl/FeCl_2_ Mixture

Herein, the acid recovery performance of the synthesized BPPO-based anion exchange membranes was revealed via a diffusion dialysis process in a two-compartment cell as reported in our previous work [35,38,39,49]. It was separated by the synthesized anion exchange membranes possessing an effective area of 5.7 cm^2^. For this, the synthesized membrane was conditioned in the feed solution (0.89 M HCl + 0.25 M FeCl_2_) for 10 h and then washed with deionized water before utilizing it in the diffusion dialysis experiment. One side of the cell was filled up with 100 mL deionized water while the other side 100 mL feed solution. After this, both side solutions were stirred vigorously to remove the concentration polarization. For each membrane sample, diffusion dialysis was performed under the same experimental conditions. At last, both feed and permeate were removed from different sides of the cell after one hour. The concentration of H^+^ on both sides was estimated by titration method while Fe^2+^ concentration was estimated inductive coupled plasma spectrophotometry (ICP, Optima 7300 DV, PerkinElmer, Waltham, MA, USA). 

The dialysis coefficients (*U*) can be determined by utilizing the below-given equation [35,49]:

(4) $$U=\frac{M}{At\Delta C}$$

In Equation (4), *M* denotes the concentration of component transported in (mol), *A* represents the effective area (m^2^) of the membrane, *t* indicates the time (h), and ∆*C* represents the logarithm average concentration between the two sides (mol/m^3^). ∆*C* was measured from the given relationship [35,50]:

(5) $$\Delta C=\frac{C_{f}^{0}-(C_{f}^{t}-C_{d}^{t})}{\ln[C_{f}^{0}/(C_{f}^{t}-C_{d}^{t})]}$$

where $C_{f}^{0}$ and $C_{f}^{t}$ are feed concentrations at time 0 and *t*, respectively, and $C_{d}^{t}$ is the dialysate.

Dialysis coefficient *U_H_* and *U_Fe_* can be measured by utilizing the above relationships (4) and (5). The separation factor (*S*) is the ratio of dialysis coefficients (*U*) of the two species present in the solution. It can be revealed from the below relationship [51,52]:

(6) $$S=\frac{U_{H}}{U_{Fe}}$$

## 3. Results and Discussions

### 3.1. Bromination of Poly (2,6-Dimethyl-1,4-Phenylene Oxide) (PPO) 

**Figure 5:**
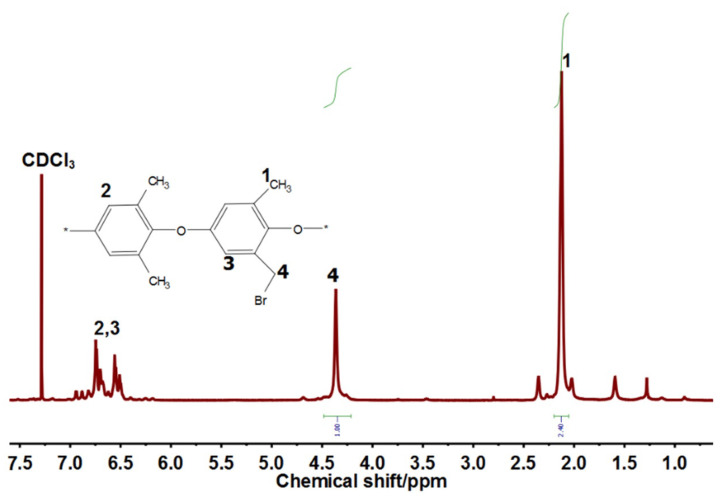
^1^H NMR spectrum of BPPO.

Bromination was done by utilizing NBS as a brominating agent and AIBN as an initiator effectively. Depending on the reaction conditions and reagents utilized in the experiments, bromination can take place either at the benzylic position or at the aromatic ring [41,53]. In this study, bromination took place effectively at benzylic position of PPO in refluxing chlorobenzene solution at 135 °C. The obtained ^1^H NMR spectrum of BPPO is represented in Figure 5. It represents that the characteristic benzyl bromide group was located at 4.3 ppm. The degree of bromination for the copolymers was calculated from the integral area ratio between the benzyl bromide group and the unreacted benzyl signal at 2.1 ppm and was stated to as 75%.

### 3.2. FTIR Spectra of the Synthesized Membrane

**Figure 6:**
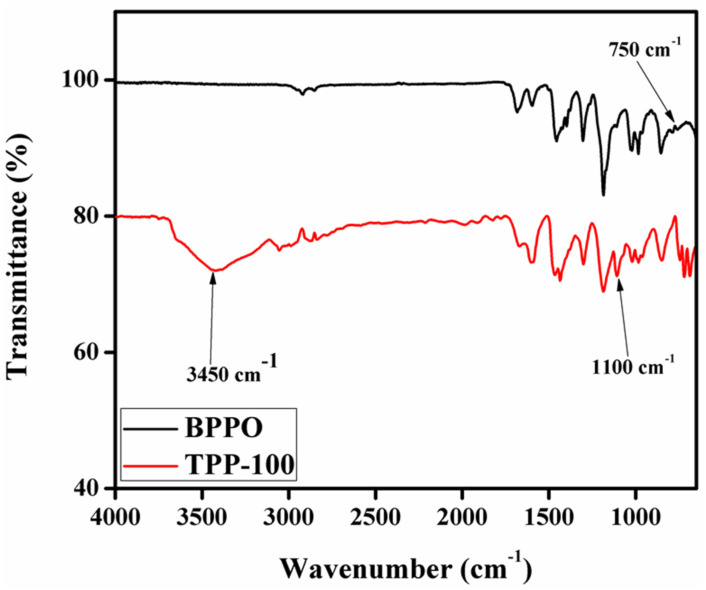
IR spectrum for pristine TPP–100 and BPPO membranes.

Herein, FTIR spectroscopy was employed to confirm whether the synthesis of BPPO-based anion exchange membranes was successful and effective or not. Figure 6 displays FTIR spectra of pure BPPO in addition to the synthesized anion exchange membrane TPP–100. The prominent bands and peaks for the BPPO membrane are displayed at 750 cm^−1^ at almost 90% transmittance and about almost 1250 cm^−1^ with almost 82% transmittance. For TPP–100 the studied peaks are at 1100 cm^−1^ with 72% transmittance and 3450 cm^−1^ with almost 74% transmittance values. The characteristic band at 750 cm^−1^ is associated with the stretching vibration of C–Br in the pure BPPO membrane [35]. The characteristic band at 1100 cm^−1^ is because of the introduction of quaternary phosphonium into the prepared anion exchange membrane TPP–100 cm^−1^. This band is not present in the pristine BPPO membrane. It shows the successful synthesis of the phosphonium quaternized anion exchange membranes. The wide and expanded band at 3450 cm^−1^ is due to the stretching vibrations of hydrogen-bonded -OH group in water molecules, which was absorbed during the FTIR analysis due to hydrophilicity of the synthesized membrane [54]. Moreover, the band at 750 cm^−1^ for C–Br stretching in bromobenzyl groups disappeared in the synthesized anion exchange membranes [35]. The successful attachment of TPP with BPPO architecture was confirmed by the above accomplished results.

### 3.3. Ion Exchange Capacity, Linear Swelling Ratio, and Water Uptake 

Ion exchange capacity, linear swelling ratio, and water uptake are some fundamental and critical parameters to conclude the performance of the membranes. Ion exchange capacity (IEC) represents the existence of exchangeable functional groups in the polymer matrix. It is crucial to control the efficiency of the prepared ion exchange membranes from diffusion dialysis process. It was measured by classical Mohr’s technique and the attained results are given in Table 1. For the prepared AEM, the value of IEC is found to be 1.22 to 1.87 mmol/g. By increasing the quantity of TPP into the polymer matrix, IEC was enhanced gradually, indicating the successful introduction of TPP into the membrane matrix with the highest IEC of TPP–100 of 1.87 and 1.22 mmol/g of TPP–43 which are very similar to [48,55,56] and the reason for this value can be the electro-static bonds that result in embedding density.

Water uptake (*W_R_*) of the ion exchange membrane demonstrates the capacity for the water hold. It depends usually on the hydrophilicity of the ion exchange membrane. For the process of diffusion dialysis for the ion exchange membrane, it is a very important and significant parameter. It aids in the movement and transference of the ions with the help of the ion exchange membrane [38,50]. Table 1 shows the water uptake percentages for the synthesized anion exchange membrane at room temperatures. The value of water uptake is found to be 44 to 67% for the prepared membrane with the highest (67%) water uptake for TPP–100. It signifies that as there is an increase in the amount of TPP into the membrane matrix, the uptake ratio for water is also enhanced from 44 to 67%. To conclude, the uptake ratio is correlated with an increase in hydrophilicity as the amount of TPP into the polymer matrix is enhanced. 

Table 1 represents the linear swelling ratio (LSR) of the formulated anion exchange membranes at room temperature. For the anion exchange membrane prepared in this study, LSR was found to be increasing drastically from 7.60 to 19.64% with increasing the amount of TPP in the membrane matrix. From this, it is summarized that the prepared anion exchange membrane possesses tremendous resistance to swelling which in other words is good for a long time run for the application of diffusion dialysis.

### 3.4. Thermal and Mechanical Stability

**Figure 7:**
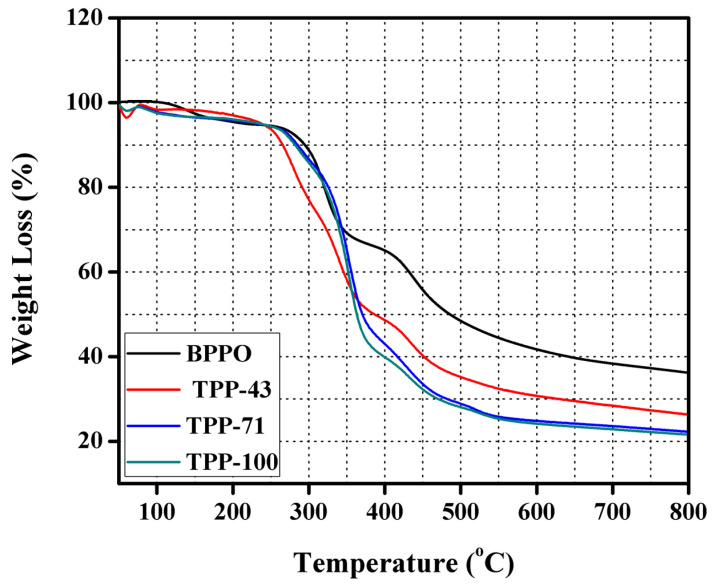
TGA thermograms of pristine BPPO and the prepared AEMs.

The prepared anion exchange membrane (TPP–43, TPP–71, and TPP–100), as well as pristine BPPO, were investigated via thermogravimetric analysis (TGA) to check and validate their thermal stabilities within the temperature range of 30 to 800 °C under nitrogen flow. Thermogravimetric analysis is basically an analysis done based on temperature through which the measurement for the mass for the sample used is taken with altering temperature and time, if there is no significant change in mass/weight, that sample is considered thermally stable. Figure 7 depicts the TGA thermogram of the prepared anion exchange membranes and pure BPPO membrane. The weight loss of the studied membranes occurred in three consecutive steps. The evaporation of surface adsorbed water from the prepared anion exchange membrane occurred around 80 to 140 °C which corresponds to the first step. Degradation of the quaternary ammonium group inside the membrane matrix took place around 180 to 250 °C which corresponds to the second step. Degradation of polymer architecture was observed around 450 °C which associates with the final weight loss step. Since with increase in temperature, the weight loss % is very low, it is concluded that the prepared BPPO-based anion exchange membranes possess thermal stability.

Table 2 shows the tensile strength (TS) and elongation at break of the prepared anion exchange membrane measured at room temperature. Tensile strength is signified as the amount of force and stress that a substance can hold upon to until it gets fragmented whereas elongation demonstrates the ductility. Tensile strength and elongation are important parameters especially in the application to test the stability of ion exchange membranes, effusive state of hydration often results in reducing the mechanical strength mainly because of the water absorption in the domains that have strong affinity with water [57,58,59].

Mechanical stability in this study investigated by utilizing a dynamic mechanical analyzer (DMA) in a wet state. Results represent that the tensile strength of the prepared anion exchange was decreased whereas elongation at break was increased with increasing the amount of TPP into the membrane matrix. The elongation at break of the prepared membranes was in the range of 14.67 to 52.20 which is lower than the reported anion exchange membranes in literature [60,61,62]. It has been observed that the anion exchange membrane with a higher elongation break possesses lower tensile strength. The prepared membrane TPP–100 with lower tensile strength and higher elongation exhibited higher flexibility. Therefore, it is concluded that the synthesized anion exchange membranes exhibited extraordinary mechanical stability with high ductility and elasticity.

### 3.5. Chemical Stability

**Figure 8:**
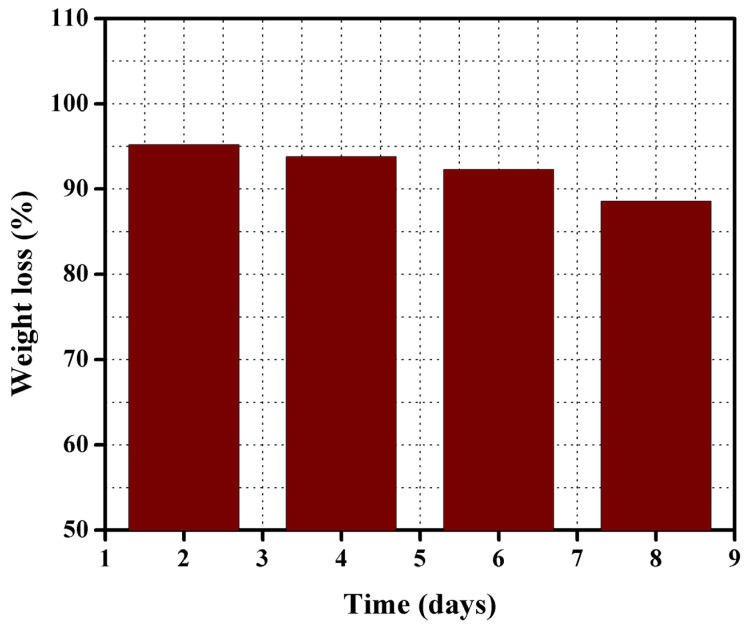
Weight loss percentage of the prepared membrane TPP–100 after immersion in HCl/FeCl_2_ feed solution for 8 days at 60 °C.

The produced membrane TPP–100 was selected to examine the chemical stability of the synthesized BPPO-based anion exchange membrane. Chemical stability in other words is the ability of a substance to resist any chemical change or reaction. The chemical stability was revealed in terms of weight loss % as a function of immersion time into the HCl/FeCl_2_ feed solution. The selected membrane TPP–100 was immersed into a feed solution for 8 days at 60 °C. Figure 8 denotes the percentage weight loss of the studied membrane as a function of immersion time. The prominent results signify that the weight loss was about 5% after 2 days, 8% after 4 days, and was only 12% after 8 days soaking into feed solution. This loss is almost negligible and hence denotes that the prepared membrane was sufficiently chemical stable. Therefore, it was concluded that the prepared anion exchange membranes are good for the recoveries of acids.

### 3.6. Membranes Morphology

**Figure 9:**
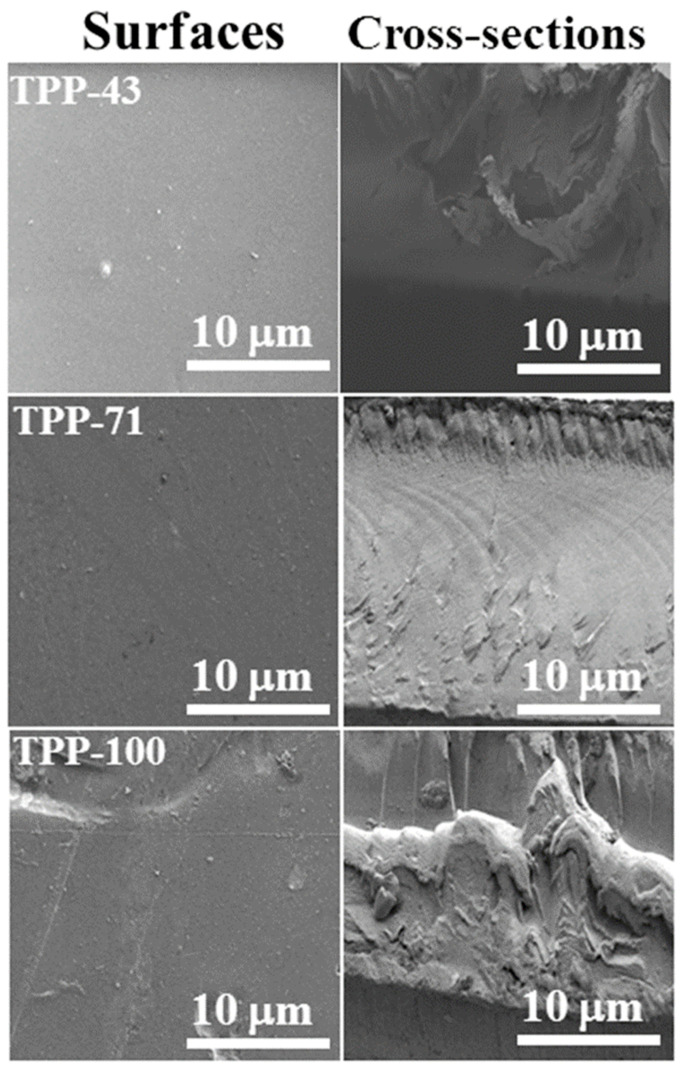
SEM micrographs of surfaces and cross-sections of the prepared membranes representing the homogeneous morphology.

Investigation for the morphological features of the formulated BPPO-based anion exchange membranes TPA–43, TPP–71, and TPP–100 was performed through the utilization of the field emission scanning electron microscope (FE-SEM, Sirion200, FEI Company Hillsboro, OR, USA). Figure 9 represents SEM micrographs of surfaces and cross-sections of the synthesized anion exchange membranes. The structure morphology that was depicted to be homogenous was validated and accepted due to the fine surface without any pores or holes in surfaces and cross-sectional views of the studied membranes. Roughness value was also very minute investigated surface and cross-sectional views of TPP–100. It can be seen that the homogeneity of the prepared AEMs was enhanced by increasing the quantity of TPP into the polymer matrix. Homogeneous morphology with fine grains and equal size distributions of anion exchange membranes are usually needed to attain higher acid recovery performance and efficiency. From this, it is concluded that the higher acid recovery via diffusion dialysis process can be achieved employing the synthesized anion exchange membranes.

### 3.7. Diffusion Dialysis for HCl/FeCl_2_ Mixture

**Figure 10:**
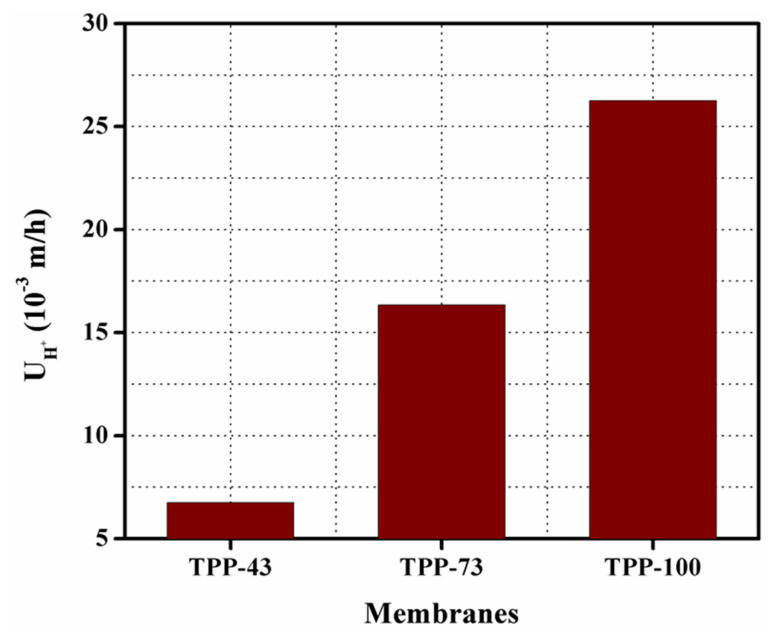
Diffusion dialysis coefficient of acid (U_H_^+^_)_ for the prepared AEMs at 25 °C.

Diffusion dialysis performance coefficient with varying the synthesized BPPO-based anion exchange membranes was studied in a two-compartment cell in batch mode, employing a mixture of HCl and FeCl_2_ as model feed at room temperature. Figure 10 shows the diffusion dialysis coefficient of acid (U_H_^+^_)_ for the prepared anion exchange membranes. The value of diffusion coefficient of acid (U_H_^+^) for the synthesized BPPO-based anion exchange membrane is found to be 6.7 to 26.3 (10^−3^ m/h) at 25 °C. It can be seen that the value of (U_H_^+^) was gradually increased from 6.7 to 26.3 (10^−3^ m/h) with enhancing the amount of TPP into the membrane matrix. Results showed that the attained value of U_H_^+^ for the prepared membrane is higher than commercial membrane DF-120B (U_H_^+^ = 0.004 m/h) [35,38,49]. Table 3 represents an interesting and unique comparison of the synthesized anion exchange membranes with membranes reported in the literature. It showed that prepared anion exchange membranes exhibited higher diffusion dialysis efficiency than the previously reported anion exchange membranes under the same experimental conditions. From this, it is summarized that the prepared anion exchange membranes have excellent potential to recover acids. The migration of ions across the anion exchange membrane is important for the diffusion dialysis process because it is responsible for the increase in U_H_^+^ of the prepared anion exchange membranes. Table 1 represents that the ion exchange capacity of the prepared anion exchange membranes was increased with increasing concentration of TPP into the polymer matrix. Thus, the prepared anion exchange membrane with high ion exchange capacity exhibits more ionic sites which support the migration of ions especially chloride ion (Cl^−^) across the membranes. The H^+^ ions may also diffuse with chloride ions across the anion exchange membrane because they have a lower valence state to satisfy the conditions of electrical neutrality [62,63,64,65,66]. Therefore, the concentration of H^+^ enhances, which leads to the increase of the U_H_^+^ value of membranes. Hence, the synthesized BPPO-based anion exchange membranes can be potentially employed for acid recovery via the diffusion dialysis process.

**Figure 11:**
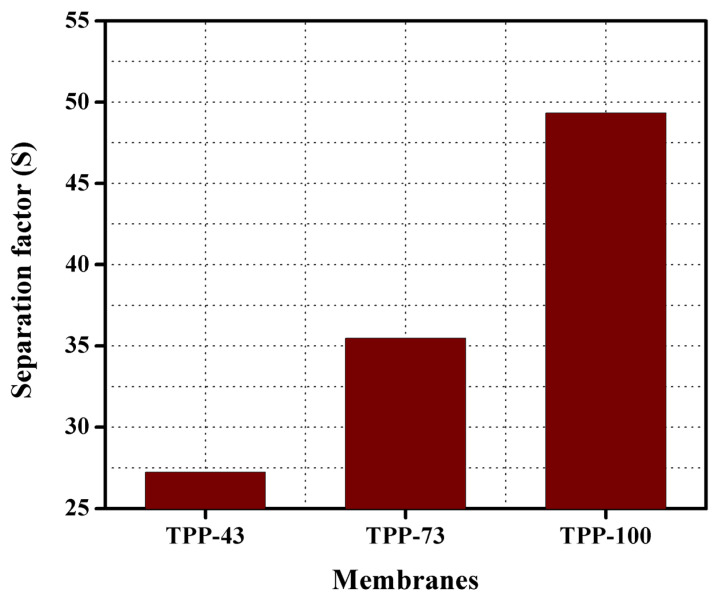
Separation factor (S) for the synthesized AEMs at temperature 25 °C.

The separation factor can also be defined as the ratio of the amount of substance that diffused with respect to what is left in the feed from a semi–permeable membrane. This separation technique merely relies on the feed concentration and the effluent concentration, because the membrane does not have sufficient selectivity, some of the unfavored substances can get transported through the membrane affecting the separation factor [42]. Figure 11 shows the separation factor (S) of the formulated anion exchange membranes TPP–43 to TPP–100 at room temperature. The reported values of S for the synthesized anion exchange membranes were in the range 27 to 49 for TPP 43–TPP 100 at room temperature. Results represented that the attained value of S is higher than commercial membrane DF–120B [38] and PVA based hybrid membrane [64,65,66,67] at 25 °C. From the membranes TPP–43 to TPP–100, the value of S is found to be enhanced from 27 to 49 with increasing the quantity of TPP into the polymer matrix. With increasing the amount of TPP into the polymer matrix, both ion exchange capacity, and water uptake of the synthesized anion exchange membrane were enhanced. It resulted to the higher value of S for the synthesized anion exchange membranes. Hence, it is concluded that in order to attain a higher value of S, an appropriate concentration of TPP is necessary for the synthesized BPPO-based anion exchange membranes at 25 °C.

## 4. Conclusions 

To conclude, a sequence of a homogenous BPPO-based anion exchange membrane has been formulated effectively with the solution casting method and is confirmed by FTIR spectroscopy. The synthesized membranes exhibited homogenous morphology with excellent thermal, mechanical, and acid stability with enhanced ion exchange capacity, linear swelling ratio, and water uptake capacity. The diffusion dialysis coefficient of acid (U_H_^+^) for the synthesized AEMs was in the range of 6.7 × 10^−3^ to 26.3 × 10^−3^ m/h whereas the factor of separation (S) was in the range of 27 to 49 at ambient conditions. Results also demonstrated that the reported value of acid recovery for the prepared AEMs in this study is comparatively better than other commercials (DF–120B) and reported membranes under the same experimental conditions. Therefore, it is highly recommended to use and to do further studies on higher significant acid recovery values and applications for the synthesized BPPO–based anion exchange membrane in this study.

## Figures and Tables

**Table 1:** Composition, theoretical ion exchange capacity (IEC_T_), experimental ion exchange capacity (IEC_Exp._), water uptake, and linear swelling ratio of prepared anion exchange membranes.

Membranes	BPPO (g)	TPP (g)	IEC_T_ (mmol/g)	IEC_Exp._ (mmol/g)	W_R_ (%)	LSR (%)
TPP–43	0.7	0.30	1.15	1.22	44	7.60
TPP–73	0.7	0.50	1.6	1.52	47	16.67
TPP–100	0.7	0.70	1.91	1.87	67	19.64

**Table 2:** Tensile strength (TS) and elongation at break of the prepared anion exchange membrane.

Membranes	TPP–43	TPP–71	TPP–100
TS (MPa)	42.70	29.05	20.87
E_b_ (%)	14.67	21.76	52.20

**Table 3:** Structure, ion exchange capacity, diffusion dialysis coefficient, and separation factor of the prepared AEMs and reported membrane employing HCl/FeCl_2_ as the model acidic waste solution at 25 °C.

Membranes	Structure	IEC (mmol/g)	U_H_^+^ (10^−3^ m/h)	S	Ref.
Quaternized BPPO–TEA membranes	Dense	1.22–1.86	6.7–26	27–49	This work
PVA based hybrid membranes	Dense	0.58–1.15	11–18	18.5–21	[63]
PVA–silica anion exchange hybrid membranes	Dense	0.52–1.01	8–10	15.9–21	[65]
Quaternized bionic multisilicon copolymers	Dense	0.46–1.25	7.20–7.50	26.9–42.8	[66]
PVA-based anion exchange hybrid membranes	Dense	0.34–0.76	10–17	12–35	[29]
Quaternized PPO based hybrid membranes	Dense	1.70–2.20	5–11	17–32	[67]
Quaternized PPO based membranes	Dense	1.10–1.80	6–18	16–28	[39]

## Data Availability

Not applicable.

## References

[1] Xue M., Kendall A., Xu Z., Schoenung J.M. (2015). Waste Management of Printed Wiring Boards: A Life Cycle Assessment of the Metals Recycling Chain from Liberation through Refining. Environ. Sci. Technol..

[2] German M., Sengupta A.K., Greenleaf J. (2013). Hydrogen Ion (H+) in Waste Acid as a Driver for Environmentally Sustainable Processes: Opportunities and Challenges. Environ. Sci. Technol..

[3] Zhang C., Zhang W., Wang Y. (2020). Diffusion Dialysis for Acid Recovery from Acidic Waste Solutions: Anion Exchange Membranes and Technology Integration. Membranes.

[4] Sharma P.P., Yadav V., Rajput A., Kulshrestha V. (2018). Poly (triethoxyvinylsilane-co-quaternaryvinylbenzylchloride)/fGNR based anion exchange membrane and its application towards salt and acid recovery. J. Membr. Sci..

[5] Barnes S., Dalhoff R., Keller J., Wilderer P., Kendall L. (2003). Investigation of membrane processes for the removal of volatile fatty acids. Water Sci. Technol..

[6] Wu L., Zhao Y., Ge L., Yang Z., Jiang C., Xu T. (2015). One-pot preparation of anion exchange membranes from bromomethylated poly(2,6-dimethyl-1,4-phenylene oxide) for electrodialysis. Chem. Eng. Sci..

[7] Tanaka N., Yamaki T., Asano M., Terai T., Onuki K. (2014). Membrane performance on electro-electrodialysis of HI–I2–H2O mixture for IS process. Nucl. Eng. Des..

[8] Salehi F., Razavi S.M., Elahi M. (2011). Purifying anion exchange resin regeneration effluent using polyamide nanofiltration membrane. Desalination.

[9] Wang P., Zhang G., Wu Y. (2015). Diffusion dialysis for separating acidic HCl/glyphosate liquor. Sep. Purif. Technol..

[10] Mao F., Zhang G., Tong J., Xu T., Wu Y. (2014). Anion exchange membranes used in diffusion dialysis for acid recovery from erosive and organic solutions. Sep. Purif. Technol..

[11] Burlakova E.B. (2007). Bioantioxidants. Russ. J. Gen. Chem..

[12] Li W., Zhang Y., Huang J., Zhu X., Wang Y. (2012). Separation and recovery of sulfuric acid from acidic vanadium leaching solution by diffusion dialysis. Sep. Purif. Technol..

[13] Wang L., Zhang F., Li Z., Liao J., Huang Y., Lei Y., Li N. (2018). Mixed-charge poly(2,6-dimethyl-phenylene oxide)anion exchange membrane for diffusion dialysis in acid recovery. J. Membr. Sci..

[14] Luo J., Wu C., Xu T., Wu Y. (2011). Diffusion dialysis-concept, principle and applications. J. Membr. Sci..

[15] Tongwen X. (2004). Tuning the diffusion dialysis performance by surface cross-linking of PPO anion exchange membranes?simultaneous recovery of sulfuric acid and nickel from electrolysis spent liquor of relatively low acid concentration. J. Hazard. Mater..

[16] Tongwen X., Weihua Y. (2001). Sulfuric acid recovery from titanium white (pigment) waste liquor using diffusion dialysis with a new series of anion exchange membranes—Static runs. J. Membr. Sci..

[17] Tongwen X., Weihua Y. (2003). Industrial recovery of mixed acid (HF + HNO3) from the titanium spent leaching solutions by diffusion dialysis with a new series of anion exchange membranes. J. Membr. Sci..

[18] Xu T., Liu Z., Huang C., Wu Y., Wu L., Yang W. (2008). Preparation of a Novel Hollow-Fiber Anion-Exchange Membrane and Its Preliminary Performance in Diffusion Dialysis. Ind. Eng. Chem. Res..

[19] Mondal A.N., Cheng C., Khan M.I., Hossain M., Emmanuel K., Ge L., Wu B., He Y., Ran J., Ge X. (2017). Improved acid recovery performance by novel Poly(DMAEM-co-γ-MPS) anion exchange membrane via diffusion dialysis. J. Membr. Sci..

[20] Afsar N.U., Erigene B., Irfan M., Wu B., Xu T., Ji W., Emmanuel K., Ge L., Xu T. (2018). High performance anion exchange membrane with proton transport pathways for diffusion dialysis. Sep. Purif. Technol..

[21] Bakangura E., Cheng C., Wu L., He Y., Ge X., Ran J., Emmanuel K., Xu T. (2016). Highly charged hierarchically structured porous anion exchange membranes with excellent performance. J. Membr. Sci..

[22] Feng J., Chen J., Wei B., Liao S., Yu Y., Li X. (2019). Series-connected hexacations cross-linked anion exchange membranes for diffusion dialysis in acid recovery. J. Membr. Sci..

[23] Liu Z., Li X., Shen K., Feng P., Zhang Y., Xu X., Hu W., Jiang Z., Guiver M.D. (2013). Naphthalene-based poly(arylene ether ketone) anion exchange membranes. J. Mater. Chem. A.

[24] Ma W., Zhao C., Lin H., Zhang G., Na H. (2011). Poly(aryl ether ketone)s with bromomethyl groups: Synthesis and quaternary amination. J. Appl. Polym. Sci..

[25] Pan J., Li Y., Han J., Li G., Tan L., Chen C., Lu J., Zhuang L. (2013). A strategy for disentangling the conductivity–stability dilemma in alkaline polymer electrolytes. Energy Environ. Sci..

[26] Li X., Yu Y., Liu Q., Meng Y. (2013). Synthesis and properties of anion conductive multiblock copolymers containing tetraphenyl methane moieties for fuel cell application. J. Membr. Sci..

[27] Li X., Yu Y., Liu Q., Meng Y. (2012). Synthesis and Properties of Anion Conductive Ionomers Containing Tetraphenyl Methane Moieties. ACS Appl. Mater. Interfaces.

[28] Chen D., Hickner M.A. (2012). Degradation of Imidazolium- and Quaternary Ammonium-Functionalized Poly(fluorenyl ether ketone sulfone) Anion Exchange Membranes. ACS Appl. Mater. Interfaces.

[29] Wu C., Wu Y., Luo J., Xu T., Fu Y. (2010). Anion exchange hybrid membranes from PVA and multi-alkoxy silicon copolymer tailored for diffusion dialysis process. J. Membr. Sci..

[30] Lin X., Varcoe J.R., Poynton S.D., Liang X., Ong A.L., Ran J., Li Y., Xu T. (2013). Alkaline polymer electrolytes containing pendant dimethylimidazolium groups for alkaline membrane fuel cells. J. Mater. Chem. A.

[31] Rebeck N.T., Li Y., Knauss D.M. (2013). Poly(phenylene oxide) copolymer anion exchange membranes. J. Polym. Sci. Part B Polym. Phys..

[32] Wang G., Weng Y., Zhao J., Chu D., Xie D., Chen R. (2009). Developing a novel alkaline anion exchange membrane derived from poly(ether-imide) for improved ionic conductivity. Polym. Adv. Technol..

[33] Li N., Guiver M.D. (2014). Ion Transport by Nanochannels in Ion-Containing Aromatic Copolymers. Macromolecules.

[34] Zhou J., Unlu M., Vega J.A., Kohl P.A. (2009). Anionic polysulfone ionomers and membranes containing fluorenyl groups for anionic fuel cells. J. Power Sources.

[35] Khan M.I., Khraisheh M., Almomani F. (2019). Fabrication and characterization of pyridinium functionalized anion exchange membranes for acid recovery. Sci. Total Environ..

[36] He Y., Pan J., Wu L., Ge L., Xu T. (2015). Facile preparation of 1,8-Diazabicyclo[5.4.0]undec-7-ene based high performance anion exchange membranes for diffusion dialysis applications. J. Membr. Sci..

[37] Lin X., Shamsaei E., Kong B., Liu J.Z., Xu T., Wang H. (2015). Fabrication of asymmetrical diffusion dialysis membranes for rapid acid recovery with high purity. J. Mater. Chem. A.

[38] Khan M.I., Mondal A.N., Emmanuel K., Hossain M., Afsar N.U., Wu L., Xu T. (2016). Preparation of pyrrolidinium-based anion-exchange membranes for acid recovery via diffusion dialysis. Sep. Sci. Technol..

[39] Khan M.I., Luque R., Prinsen P., Rehman A.U., Anjum S., Nawaz M., Shaheen A., Zafar S., Mustaqeem M. (2017). BPPO-Based Anion Exchange Membranes for Acid Recovery via Diffusion Dialysis. Materials.

[40] Ji W., Wu B., Zhu Y., Irfan M., Afsar N.U., Ge L., Xu T. (2020). Self-organized nanostructured anion exchange membranes for acid recovery. Chem. Eng. J..

[41] Li N., Yan T., Li Z., Thurn-Albrecht T., Binder W.H. (2012). Comb-shaped polymers to enhance hydroxide transport in anion exchange membranes. Energy Environ. Sci..

[42] Kadłubowicz A., Janiszewska M., Baraniak M., Lota G., Staszak K., Regel-Rosocka M. (2021). Diffusion dialysis and extraction integrated system for recovery of cobalt(II) from industrial effluent. J. Water Process. Eng..

[43] Khan M.I., Khraisheh M. (2018). Synthesis and characterization of stable anion exchange membranes for desalination applications. Desalination Water Treat..

[44] Khan M.I. (2018). Comparison of different quaternary ammonium groups on desalination performance of BPPO-based anion exchange membranes. Desalination Water Treat..

[45] Khan M.I., Li X., Fernandez-Garcia J., Lashari M.H., Rehman A.U., Elboughdiri N., Kolsi L., Ghernaout D. (2021). Effect of Different Quaternary Ammonium Groups on the Hydroxide Conductivity and Stability of Anion Exchange Membranes. ACS Omega.

[46] Khan M., Shanableh A., Fernandez J., Lashari M., Shahida S., Manzoor S., Zafar S., Rehman A.U., Elboughdiri N. (2021). Synthesis of DMEA-Grafted Anion Exchange Membrane for Adsorptive Discharge of Methyl Orange from Wastewaters. Membranes.

[47] Khan M.I., Khraisheh M., Buzdar A.R., Munir Khan M.U., Rehman A., Prapamonthon P., Hassan W., Aziz A., Farooq M. (2018). Development and surface modification of anion exchange membrane for enhancement of antifouling potential in electrodialysis process. Fresenius Environ. Bull..

[48] Khan M.I., Zheng C., Mondal A.N., Hossain M., Wu B., Emmanuel K., Wu L., Xu T. (2017). Preparation of anion exchange membranes from BPPO and dimethylethanolamine for electrodialysis. Desalination.

[49] Khan M.I., Mondal A.N., Cheng C., Pan J., Emmanuel K., Wu L., Xu T. (2016). Porous BPPO-based membranes modified by aromatic amine for acid recovery. Sep. Purif. Technol..

[50] Khan M.I., Su J., Lichtfouse E., Guo L. (2020). Higher efficiency of triethanolamine-grafted anion exchange membranes for acidic wastewater treatment. Desalination Water Treat..

[51] Khan M., Shanableh A., Elboughdiri N., Kriaa K., Ghernaout D., Ghareba S., Khraisheh M., Lashari M. (2021). Higher Acid Recovery Efficiency of Novel Functionalized Inorganic/Organic Composite Anion Exchange Membranes from Acidic Wastewater. Membranes.

[52] Khan M.I., Su J., Guo L. (2021). Preparation and characterization of high performance anion exchange membranes for acid recovery. Desalination Water Treat..

[53] Tongwen X., Weihua Y. (2001). Fundamental studies of a new series of anion exchange membranes: Membrane preparation and characterization. J. Membr. Sci..

[54] Hossain M., Wu L., Liang X., Yang Z., Hou J., Xu T. (2018). Anion exchange membrane crosslinked in the easiest way stands out for fuel cells. J. Power Sources.

[55] Khan M.I., Mondal A.N., Tong B., Jiang C., Emmanuel K., Yang Z., Wu L., Xu T. (2016). Development of BPPO-based anion exchange membranes for electrodialysis desalination applications. Desalination.

[56] Zhang P., Wu Y., Liu W., Cui P., Huang Q., Ran J. (2021). Construction of two dimensional anion exchange membranes to boost acid recovery performances. J. Membr. Sci..

[57] Chu J.Y., Lee K.H., Kim A.R., Yoo D.J. (2020). Improved electrochemical performance of composite anion exchange membranes for fuel cells through cross linking of the polymer chain with functionalized graphene oxide. J. Membr. Sci..

[58] Lin B., Xu F., Chu F., Ren Y., Ding J., Yan F. (2019). Bis-imidazolium based poly(phenylene oxide) anion exchange membranes for fuel cells: The effect of cross-linking. J. Mater. Chem. A.

[59] Lee K.H., Chu J.Y., Kim A.R., Yoo D.J. (2018). Enhanced Performance of a Sulfonated Poly(arylene ether ketone) Block Copolymer Bearing Pendant Sulfonic Acid Groups for Polymer Electrolyte Membrane Fuel Cells Operating at 80% Relative Humidity. ACS Appl. Mater. Interfaces.

[60] Emmanuel K., Cheng C., Erigene B., Mondal A.N., Afsar N.U., Khan M.I., Hossain M., Jiang C., Ge L., Wu L. (2017). Novel synthetic route to prepare doubly quaternized anion exchange membranes for diffusion dialysis application. Sep. Purif. Technol..

[61] Emmanuel K., Erigene B., Cheng C., Mondal A.N., Hossain M., Khan M.I., Afsar N.U., Ge L., Wu L., Xu T. (2016). Facile synthesis of pyridinium functionalized anion exchange membranes for diffusion dialysis application. Sep. Purif. Technol..

[62] Mondal A.N., Cheng C., Yao Z., Pan J., Hossain M., Khan M.I., Yang Z., Wu L., Xu T. (2015). Novel quaternized aromatic amine based hybrid PVA membranes for acid recovery. J. Membr. Sci..

[63] Cheng C., Yang Z., Pan J., Tong B., Xu T. (2014). Facile and cost effective PVA based hybrid membrane fabrication for acid recovery. Sep. Purif. Technol..

[64] Wu Y., Luo J., Zhao L., Zhang G., Wu C., Xu T. (2013). QPPO/PVA anion exchange hybrid membranes from double crosslinking agents for acid recovery. J. Membr. Sci..

[65] Wu Y., Wu C., Li Y., Xu T., Fu Y. (2010). PVA–silica anion-exchange hybrid membranes prepared through a copolymer crosslinking agent. J. Membr. Sci..

[66] Wu Y., Luo J., Wu C., Xu T., Fu Y. (2011). Bionic Multisilicon Copolymers Used As Novel Cross-Linking Agents for Preparing Anion Exchange Hybrid Membranes. J. Phys. Chem. B.

[67] Luo J., Wu C., Wu Y., Xu T. (2010). Diffusion dialysis of hydrochloride acid at different temperatures using PPO–SiO2 hybrid anion exchange membranes. J. Membr. Sci..

